# The psychological influencing factors for the elderly in using mobile applications and the mechanism of user experience

**DOI:** 10.3389/fpsyg.2025.1609302

**Published:** 2025-07-18

**Authors:** Shining Jin

**Affiliations:** School of Design, Fujian University of Technology, Fuzhou, Fujian, China

**Keywords:** mobile applications, user experience, cognitive psychology, factor analysis, indicators of evaluation

## Abstract

**Objective:**

This study is purposed to assess the user experience factors of elderly mobile applications from the cognitive psychology perspective and establish a comprehensive evaluation index system for the elderly-friendly experience of mobile applications.

**Methods:**

The research subjects were the elderly. Their basic information, mobilephone usage experience, and demands for elderly-friendly experience were collected via questionnaires. The questionnaire design with a focus on the cognitive capabilities of the elderly as well as their emotional responses. Factor analysis was employed to process the questionnaire data, and three main factors were extracted: perceivability, operability, and comprehensibility. Additionally, the actual usage of two elderly-friendly mobile applications (WeChat and Toutiao Big Font Version) was evaluated through task tests. The task tests involved tasks such as mode switching, information viewing, and completion of specific operations.

**Results:**

The research findings demonstrate that perceptibility, operability, and comprehensibility are the principal factors affecting the mobile application experience of elderly users. Specifically, factors such as adjusting the font size of the interface, increasing the line spacing and character spacing of paragraph text, and providing images and text descriptions on the interface have a significant impact on the user experience. For instance, larger fonts and clear icon designs can significantly enhance the information perception ability of the elderly and reduce visual fatigue. Simple gesture operations and sufficient response time contribute to improving operability and resolving the operational difficulties resulting from the elderly’s motor skill limitations. Furthermore, the research also discovered that the age of elderly users have varying degrees of influence on the evaluation indicators of elderly-friendly design.

**Conclusion:**

This study, starting from the cognitive psychology perspective, proposes a scientific evaluation framework for elderly-friendly design, emphasizing the significance of considering the cognitive abilities and emotional needs of elderly users in the design process. The research results provide a theoretical foundation for the design and improvement of mobile applications, facilitating the enhancement of the usage experience and satisfaction of elderly users. Future research can further expand the sample range, conduct long-term follow-up studies, and integrate emerging technologies to optimize elderly-friendly design.

## 1 Introduction

Aging has become a global concern. The proportion of the world’s population aged 65 and above is continuously increasing, and it is projected that by 2050, this proportion will approach 16%, equivalent to over 1.5 billion people (Leeson, 2018). In the context of an aging population, the quality of life for older adults extends beyond physical health, encompassing multiple dimensions such as psychological wellbeing, satisfaction in social interactions, and overall life satisfaction (Parra-Rizo et al., 2022). Particularly, with the advancement of technology in the digital age, the digital divide among the elderly has become a governance challenge in the development of digital information. Compared with other age groups, the elderly are at a disadvantage in the digital divide (Jimoyiannis and Gravani, 2014; Mitchell et al., 2019). However, in the digital age, smartphones have become indispensable tools in people’s lives, and mobile applications are part of the daily lives of the elderly. However, the proportion of the elderly using new technologies is significantly lower than that of other age groups (Barbosa and Frank, 2019). Therefore, an increasing number of people have realized that helping the elderly bridge the digital divide and enhancing their ability to use digital technologies such as mobile applications plays an indispensable role in improving their quality of life.

How to integrate technology into the daily lives of the elderly has become a key issue (Iancu and Bogdan, 2020). Currently, many mobile applications have developed versions for elderly users. As the elderly’s physiological functions age, it is reflected in the aging of sensory functions, which mainly affects the use of internet devices and mobile applications in terms of vision and hearing. Visual aging is mainly manifested in the decline of long-distance perception ability, adaptation to darkness and glare, and the ability to distinguish and contrast colors (Stephanie Morey et al., 2017). The decline in the elderly’s memory makes it difficult for the brain to encode new information and retrieve information from long-term memory, mainly affecting the recall and recognition functions in cognition. However, recall is much more difficult because it requires users to retrieve relevant information and knowledge from long-term memory (Nilsson, 2003). Modern information interaction technologies have not been stored in the memories of the elderly, and they need to spend more time learning new technologies, making it more difficult for them to accept and use new technologies, which has a negative impact on their experience of using mobile applications (Holzinger et al., 2007; Baharum et al., 2017).

Although many studies have proposed various user experience (UX) design principles, few have comprehensively discussed the importance of these factors in the actual operation of mobile interfaces by the elderly (Ho and Tzeng, 2021; Kim et al., 2019; Huang et al., 2024). Therefore, the goal of this study is to provide design improvement directions and reliable basis for elderly versions of mobile applications through in-depth analysis of the evaluation of their usage experience.

However, there remains a notable gap in the comprehensive and in-depth investigation of the significance of these factors within the actual operation of mobile interfaces by elderly users. Consequently, this study endeavors to conduct an in-depth analysis of elderly users’ evaluation of mobile application usage experience from the perspective of cognitive psychology. It seeks to identify the critical factors influencing their experience and to offer scientifically grounded and reliable basis for elderly versions of mobile applications through in-depth analysis of the evaluation of their usage experience, thereby effectively enhancing the quality of life for the elderly.

## 2 Literature review

### 2.1 User experience

User experience (UX) is closely intertwined with psychology. It pertains to people’s perception and response toward products, systems, or services they utilize or anticipate using, yet its definition remains ambiguous (Law et al., 2014). Currently, researchers have identified two limitations in evaluating UX: one is the difficulty in quantification, and the other is the dearth of systematic approaches to assess UX (Park et al., 2013). These limitations are also present in the evaluation of elderly-friendly experience design. Numerous scholars have endeavored to delineate the elements of UX design by delving into its dimensions. UX encompasses the manner in which materials are perceived, adaptability, the user’s comprehension level, and the sensations during usage. Consequently, it can mainly be categorized into two aspects: objective material experience and subjective feeling experience (Forlizzi and Ford, 2000).

On one hand, UX research primarily unfolds around the design dimensions of human-computer interaction behavior. The stage of experience behavior is hierarchically classified as action goals, task goals, and completion goals. On this basis, the concept of usage patterns is put forward. In the interaction process, the usage patterns of scene factors can be divided into goal patterns and behavior patterns. The former centers on achieving the goal and is triggered from the outset of attaining the goal; the latter pivots on behavior and is initiated from the action goal and task goal (Hassenzahl, 2010). Actions are triggered by the action goal, thereby achieving the task goal and fulfilling the user, and the ultimate completion goal aligns with the initial requirement, thereby attaining the user’s pleasure (Pucillo and Cascini, 2014).

On the other hand, UX research focuses on the design dimensions of user emotions. Hedonic quality is proposed as a complement to the quality of human-computer interaction products. On this basis, the cognitive component is regarded as information processing within the experience dimension. Emotional responses and emotional outcomes interact with the cognitive component (Hassenzahl, 2004). Simultaneously, aspects related to non-utilitarian experience quality can be subsumed into three indicators: enjoyment, aesthetics, and pleasure (Mahlke, 2005). Therefore, it is ascertained that a favorable UX design encompasses three dimensions: aesthetic experience, functional experience, and emotional experience. Nevertheless, these three dimensions are arduous to measure when evaluating elderly UX design. Elderly user experience responses can influence cognition and also impact the evaluation and behavior resulting from cognition. Transform the measurement of elderly UX into satisfaction, usability, and emotional response (Hu et al., 2020).

### 2.2 UX factors for elderly mobile applications

The rapid advancement of mobile technology has rendered people increasingly reliant on mobile applications in their daily activities. Nevertheless, the designs of these applications frequently neglect the distinctive requirements of the elderly, giving rise to suboptimal user experiences. A multitude of empirical studies have offered valuable insights into the practical application of UX evaluation methods. For example, conducted an evaluation of the UX of elderly health management applications, emphasizing the significance of large fonts, simple navigation, explicit instructions, and error prevention functionalities (Czaja et al., 2019; Mitzner et al., 2010). Some research has identified the key UX factors that significantly influence the usability of mobile applications for the elderly. For instance, font size, line spacing, and color contrast are of paramount importance for perceptibility, as the elderly often suffer from visual impairments (Hsieh et al., 2022). Similarly, simple gestures and adequate operation time enhance operability and address the motor skill limitations of this cohort. To systematically assess the UX of mobile applications for the elderly, researchers have adopted diverse evaluation approaches, including factor analysis. Factor analysis facilitates the reduction of data dimensionality and the identification of the most crucial factors affecting UX (Hair, 2009). Utilizing factor analysis to extract the key UX factors from a plethora of variables provides a more targeted evaluation framework (Perrig et al., 2024). Research indicates that personal characteristics such as age, gender, and emotional state can profoundly impact the UX of mobile applications for the elderly. For instance, the elderly in the 60–64 age group may have distinct preferences and capabilities compared to those above 70 (Czaja et al., 2019). Gender disparities have also been noted, with men and women potentially exhibiting different levels of comfort and proficiency with technology (Hunsaker and Hargittai, 2018).

### 2.3 Cognitive factors affecting mobile applications in the elderly

The evaluation of elderly UX design can be categorized into explicit assessment and implicit assessment. Explicit assessment primarily centers on the visual interaction and traditional interface form design level. Elderly individuals encounter significant difficulties when performing interaction operations such as clicking, sliding, scrolling or flipping (Harada et al., 2013), and also face certain challenges in the aspect of selecting menus and content areas through touch interaction in mobile application products. Some aspects related to the cognitive abilities of the elderly and the processing of interaction information have been largely overlooked, particularly with respect to information navigation and the usability design of iconography (Petrovčič et al., 2018). Studies on the relationship among the usage behavior of new technologies by elderly users, user perception, and user characteristics have revealed that navigation issues and icon design are the principal aspects influencing the perceived usability among the elderly (Li and Luximon, 2018). Implicit assessment focuses on the mental health, acceptance, and wellbeing of the elderly. Some scholars have endeavored to dissect the research strategies and methods of design from distinct design perspectives, such as matching the cognitive abilities of the elderly, eliminating stereotypes regarding the elderly, and behavioral adaptability. Through wearable watches, they have analyzed the stereotypes about the elderly in design, which are mainly manifested in the lack of aesthetic appeal suitable for the elderly, unoptimized functional disparities, and neglect of privacy, thereby providing a basis for the improvement of designs for the elderly (Li et al., 2020). Cognitive psychology approaches are employed to evaluate the cognitive abilities of the elderly, such as spatial awareness, memory, and comprehension, and design cases are utilized to verify the degree of matching between user capabilities and design requirements (Li, 2019).

Although the UX indicators of mobile interfaces exhibit a certain degree of universality, there are scarce elderly experience evaluation indicators that are entirely applicable to mobile applications specifically designed for the elderly. Hence, a key issue emerges: In mobile applications dedicated for the elderly, how is the elderly UX evaluated, and how is the significance of design elements for the elderly distributed?

## 3 Research framework

**FIGURE 1:**
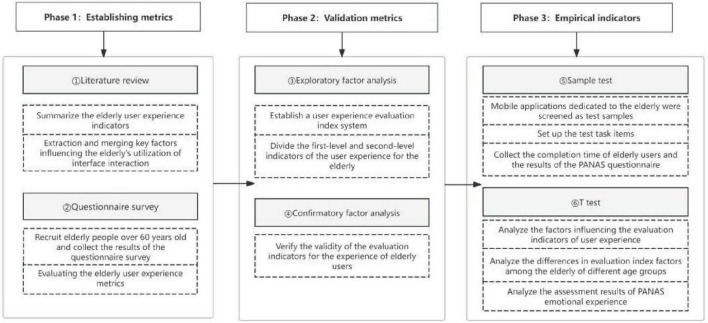
The overall research framework of this study.

The overall research framework of this study, as depicted in Figure 1, encompasses three primary phases: establishing indicators, validating indicators, and conducting empirical tests of indicators.

In the first phase, literature analysis is employed to collate relevant research and technical standards regarding the interface design of mobile applications and the evaluation of elderly user experiences, and to construct evaluation indicators for elderly users’ experiences. Through questionnaire surveys of the evaluation results of elderly users’ experiences with mobile applications, the evaluation indicator system for elderly mobile application experiences is determined.

The second phase, which pertains to the validation of evaluation indicators, involves the application of statistical techniques. Factor analysis is adopted to delineate the hierarchical relationships of evaluation indicators, calculate the scores and weight rankings of each factor, and explore the preference requirements of indicator factors for the evaluation of elderly mobile application experiences.

In the final phase, three mobile applications specifically developed for elderly users are utilized as samples. The durations taken by elderly users to complete various indicator tasks are collected to obtain feedback results on the evaluation indicators of elderly user experiences, thereby ascertaining the relative significance of these evaluation indicator factors. Subsequently, *t*-tests are employed to examine the disparities related to the evaluation of elderly user experiences

## 4 The selection process of evaluation indicators

### 4.1 Establishment of evaluation indicators

Firstly, the research conducted a literature review to collate the key factors influencing the elderly’s utilization of interface interaction, as presented in Table 1. The evaluation system encompasses the target layer, first-level indicators, and second-level indicators. Among them, the first-level indicators comprise three metrics:perceptibility, operability, and comprehensibility. The second-level indicators encompass 12 indicators such as adjustable font size, increased line and character spacing of paragraph text, the necessity of images and textual explanations on the interface, provision of color alerts, setting of floating window quick control elements on the interface, simple gesture operations, offering sufficient operation time, rapid search for content, categorization of commonly used application functions, confirmation and consent for login and usage, confirmation of mobile phone verification code information, and setting a version for elderly users. This constitutes the indicator system for elderly-friendly design of mobile applications.

**TABLE 1:** Abbreviated study survey.

No.	Category	Factor definition	References
1	The adjustability of font size	The font size and font type can be adjusted.	Hou and Hu, 2023; Lin and Ho, 2020; Czaja et al., 2019
2	The increase of the line spacing and paragraph spacing of the text	Adjustable font line spacing and character spacing	Hou and Hu, 2023; Lin and Ho, 2020; Czaja et al., 2019) Hsieh et al., 2022
3	The interface demands images and textual descriptions	The interface design combines icons and text.	Czaja et al., 2019
4	Interface color reminder	It can provide information alerts for errors, actions, and confirmations.	Salazar-Cardona et al., 2023
5	The interface setting of the floating window quick control	It sets up buttons that are easy to open windows.	Salazar-Cardona et al., 2023; Nielsen, 1994; Menghi et al., 2017
6	Simple gesture operations	It allows operations through clicking, dragging, and swiping.	Nielsen, 1994; Menghi et al., 2017
7	The provision of sufficient operation time	It provides sufficient operation time.	Nielsen, 1994; Brunzini et al., 2022
8	The rapid search for content	.It offers a quick search function to directly access content.	Wildenbos et al., 2019; Czaja et al., 2019
9	The categorization of frequently used application functions	It categorizes the spatial positions of frequently used application functions for users.	Ghorbel et al., 2017
10	The confirmation and agreement to log in and use	User consent is required for login and use	Wildenbos et al., 2019; Iqbal et al., 2020
11	The confirmation of mobile phone verification code information	It sends mobile phone verification codes for information confirmation.	Wildenbos et al., 2019; Iqbal et al., 2020
12	The setting of the version for elderly users to use	It provides a special version for elderly users.	Iqbal et al., 2020; McLaughlin and Pak, 2020; Fischer et al., 2020

Based on the measurement items listed in Table 1, a questionnaire on the factors of elderly interface interaction experience was designed. Subsequently, a questionnaire survey and test were administered to elderly users. The questionnaire survey consists of 2 components: The first component pertains to the basic demographic information of the elderly, their experience in using mobile phones, the functions of mobile applications they employ, and the application of elderly-friendly modifications. The second component involves the elderly users’ assessment of the significance of the experience of mobile applications, utilizing a 5-point Likert scale. Considering the physical and mental constraints of the elderly, the process of filling out the questionnaire takes approximately 15 min. Now, the two components of the questionnaire will be expounded. For instance, Item 1 “The adjustability of font size” corresponds to the question in the questionnaire: “When interacting with the existing mobile interface for the elderly, special attention should be paid to the style, size and spacing Settings of the text to ensure a good user experience.”

### 4.2 Data collection

A total of 50 questionnaires were distributed in this pre-test, and 45 were retrieved. After eliminating the questionnaires with all identical responses, a total of 35 valid questionnaires remained. Based on the overall reliability and partial item statistical indicators, the internal consistency of the evaluation scale and question options was evaluated. The results of this pre-test questionnaire survey were also employed as the formal questionnaire.

Recruiting a large-scale random sample from a specific age group (60 years old and above) for the questionnaire survey poses challenges. Thus, this study employed a convenience sampling approach. When the research aim is of a preliminary or exploratory nature, the convenience sampling method proves highly valuable. This is because it obviates the need for a large-scale and intricate sample while still offering initial insights. Prior to administering the questionnaire, detailed information regarding the research purpose, privacy protection, and data utilization was furnished to the participants. Informed consent was solicited, and participants were required to sign the informed consent form to ensure that they comprehended and consented to participate in the study.

A total of 50 questionnaires were distributed in this preliminary assessment, and 45 were retrieved. After excluding questionnaires with identical responses, 35 valid questionnaires remained. Based on the overall reliability and select item statistics, the internal consistency level (Cronbach’s alpha coefficient) of the questionnaire scale fell within the range of 0.70–0.92. The internal consistency of the rating scale and question options was evaluated. The results of the pre-test questionnaire survey were also adopted as the formal questionnaire.

When the research objective is preliminary or exploratory, does not require a large-scale and complex sample, and can still provide preliminary insights, the convenience sampling method is highly useful. Elderly individuals aged 60 and above were recruited, and an offline survey method was adopted, with the questionnaire distribution period lasting approximately 1 month. The questionnaire distribution lasted for approximately 1 month. A total of 170 questionnaires were collected, with 158 valid questionnaires ultimately obtained, resulting in an effective questionnaire recovery rate of 92.92%. The sample size met the requirements for factor analysis (Comrey and Lee, 1992). The demographic information of the participants is presented in Table 2. Among them, the proportion of elderly respondents who have used mobile phones for 1–3 years is the highest at 44.3%, while those who have used them for less than 1 year and more than 3 years have approximately the same proportion. 40.5% of the elderly respondents use their mobile phones for an average of 1–3 h per day. 76.5% of the elderly respondents have sought assistance from others through mobile applications. 22.2% of the elderly respondents indicated that they have encountered problems when using mobile phones for medical purposes, accounting for the largest proportion. 46.3% of the elderly respondents have used the elderly-friendly versions of mobile applications but did not continue to use them.

**TABLE 2:** The demographic information of the respondents.

Sample	Category	Number	Percentage (%)
Gender	Male	84	53.1
	Female	74	46.9
Age	60–64	85	53.8
	65–69	49	31.0
	Over70	24	15.2

The structural validity of the questionnaire data was analyzed via the KMO value and Bartlett’s sphericity test to determine the applicability of factor number analysis. The test results indicated that the KMO value of this questionnaire was 0.868, suggesting a favorable correlation among the factors. The Bartlett’s sphericity test was less than 0.05, signifying that the data of this survey was applicable for factor analysis [43].

### 4.3 Quantitative evaluation indicators

#### 4.3.1 Analysis of evaluation indexes

The factor loading coefficients can be utilized to gauge the correspondence between common factors and factor variables. In conjunction with the rotation of the maximum variance method, the correlation between common factors and factor variables can be analyzed. Employing SPSS 26.0 to undertake exploratory factor analysis on the questionnaire data, a total of three common factors with eigenvalues greater than 1 were acquired, and the cumulative explanatory rate of the three common factors reached 71.641%. The specific outcomes are presented in Table 3. Based on the cumulative explanatory rate and eigenvalues, three common factors can be selected as the first-level indicator layer for delineating and expounding the evaluation of the elderly-friendly experience design of mobile applications. The indicator loadings corresponding to common factors F1, F2, and F3 are all greater than 0.5. Hence, the 12 evaluation indicators set in the questionnaire are classified into three common factors: common factor 1 is perceptibility (F1), common factor 2 is understandability (F2), and common factor 3 is operability (F3). Each indicator combination exhibits distinct characteristics, and the three generated common factors have extremely weak correlations with one another, with virtually no information duplication. This implies that the evaluation index system for the elderly-friendly design of mobile applications is scientifically rational, and the evaluation index system for the elderly-friendly design of mobile applications has been clearly established.

**TABLE 3:** The total variance explained.

Factor	Eigenvalue	% of variance	Cumulative%
F1	3.924	32.702	32.702
F2	2.353	19.609	52.311
F3	2.32	19.33	71.641

#### 4.3.2 Weights of evaluation indicators

The hierarchical relationship of the evaluation index system for the elderly mobile application experience design. In the first-level index layer, three common factors were extracted by applying the factor analysis method. According to the fundamental principle of factor analysis, the factor score coefficient matrix is the coefficient matrix of the linear combination of each variable of the principal component obtained through mathematical transformation in the factor analysis process. The specific results are presented in Table 4.

**TABLE 4:** Experience indicators for elderly mobile applications.

Indicators factors	Item	Factor loading quantity			Score coefficient	Weight	Pos
		F1	F2	F3			
Eigenroots (after rotation)		3.924	2.353	2.32			
Coefficient of determination		32.70%	19.61%	19.33%			
Perceptibility	Q1	0.4105	0.106	0.0852	0.2394	8.55%	5
	Q2	0.4093	0.1091	0.1016	0.2441	8.72%	3
	Q3	0.3916	0.1181	0.0837	0.2337	8.34%	6
	Q4	0.3928	0.1047	0.0756	0.2284	8.15%	8
	Q10	0.3812	0.1347	0.1074	0.2399	8.56%	4
	Q11	0.3654	0.2211	0.1123	0.2576	9.20%	1
Operability	Q6	0.1808	0.5089	0.109	0.2512	8.97%	2
	Q7	0.1208	0.5448	0.1068	0.2331	8.32%	7
	Q5	0.0909	0.5416	0.1425	0.2282	8.15%	9
Comprehensibility	Q12	0.0743	0.1283	0.5615	0.2205	7.87%	10
	Q9	0.1121	0.0812	0.5451	0.2205	7.87%	11
	Q8	0.0619	0.1128	0.538	0.2043	7.29%	12

Based on the factor analysis method, the dimensionality reduction of data and indicators is highly efficient, addressing the issues of overlap and correlation among indicators and ensuring the credibility and objectivity of the indicators. In the corresponding second-level index layer, perceivability encompasses six indicator factors, among which the confirmation of mobile phone verification code information and increasing the line spacing and character spacing of paragraph text rank first and third respectively; operability and understandability each comprise three indicator factors, among which the weight of simple gesture operation ranks second.

This study employs confirmatory factor analysis as a tool to assess the measurement model, ensuring the validity and discriminant validity of the measurement model. The main objective of confirmatory factor analysis is to evaluate the relationship between factors and measurement items to ensure a satisfactory association between each item and its corresponding factor. The average variance extracted (AVE) is calculated to measure the discriminant validity of each factor. Additionally, to further evaluate the model fit, we examined the composite reliability (CR) values, as shown in Table 5. It was observed that all standardized loading coefficients exceeded 0.6 (Shevlin and Miles, 1998), indicating a significant correlation between the measurement items and their corresponding factors. Simultaneously, the AVE values exceeded 0.5 (Fornell and Larcker, 1981), and the CR values exceeded 0.8, further confirming the internal consistency of the measurement items within each factor (Ahmad et al., 2016). Therefore, our research findings suggest that the measurement model has satisfactory convergent validity.

**TABLE 5:** Analysis results of convergent validity.

Factor	Code	Unstd.	S.E	CR	P	Std	AVE	CR
Perceptibility	Q1	1	–	–	–	0.789	0.599	0.899
	Q2	0.927	0.085	10.847	0	0.804		
	Q3	0.909	0.09	10.119	0	0.759		
	Q4	0.96	0.097	9.93	0	0.748		
	Q10	0.929	0.092	10.118	0	0.759		
	Q11	1.028	0.098	10.495	0	0.782		
Operability	Q6	1	–	–	–	0.845	0.652	0.849
	Q7	1.017	0.095	10.757	0	0.806		
	Q5	0.913	0.089	10.27	0	0.77		
Comprehensibility	Q12	1	–	–	–	0.856	0.632	0.837
	Q9	0.947	0.095	9.949	0	0.79		
	Q8	0.805	0.086	9.356	0	0.735		

To evaluate the discriminant validity among the factors, we calculated the square root of AVE, as presented in Table 6. The results of the confirmatory factor analysis demonstrated the robustness of our measurement model and verified the validity and discriminant validity of the measurement items within each factor.

**TABLE 6:** Analysis results of discriminant validity.

Factor	Factor 1	Factor 2	Factor 3
	Perceptibility	Operability	Comprehensibility
Perceptibility	0.774		
Operability	0.548	0.808	
Comprehensibility	0.398	0.433	0.795

## 5 Empirical study of evaluation indicators

### 5.1 The completion state of sample tasks

Based on the findings of the questionnaire survey, WeChat and Toutiao Big Font Version are the mobile applications that are most frequently utilized by the elderly respondents, with the proportions of the respondents being 44.7 and 35.23% respectively. These two mobile applications serve as the task samples in this study.

Consequently, two selected mobile applications are analyzed initially. Firstly, with respect to the elderly UX, the elderly usage mode of WeChat is incorporated within the existing mobile application version, requiring a switch to the elderly mode without the need for re-downloading. On the contrary, Toutiao Big Font Version has specifically developed and operates an independent elderly-friendly version of the application, which demands re-downloading, namely Toutiao Big Font Version. Toutiao Big Font Version possesses more pronounced elderly-friendly characteristics, such as enlarged fonts, simplified interface information content, and the addition of the function of listening to articles. The contents of the task tests for evaluating the elderly mobile application experience were formulated. First, the tasks of WeChat consist of three items: switching to the care mode, checking the itinerary code, and shopping through mini-programs. Second, the tasks of Toutiao Big Font Version consist of two items: viewing hot news and listening to news.

In this test was conducted in the elderly classroom of the community service center, including the mobile application task test and the PANAS emotional experience assessment. A total of 22 elderly people were recruited for the test. The usage duration of each task was collected through mobile phone screen recording. Based on the evaluation index points for the elderly UX, the completion durations of the task content labels for WeChat and Toutiao Big Font Version were obtained. The durations were multiplied by the index weights to separately calculate the maximum, minimum, average, and standard deviation of the experience evaluation indicators of these two mobile applications. As these two mobile applications have their respective functional attributes, not all data for the experience evaluation indicator factors could be collected. Hence, for the scoring results of the evaluation indicators that were not generated, they are indicated by “–.”

• WeChat

ConclIt can be observed from Table 7 that the score of operability within the factor layer of the elderly UX evaluation index is higher than the other two indicators. In the task of evaluating the elderly users’ experience of using WeChat, it was discovered that the three evaluation indicators of simple gesture operation, adjustable font size, and increased line spacing and paragraph spacing of text were relatively high. Specifically, it is manifested in the quick pull-down tab navigation operation during shopping in mini-programs and the barrier-free interface display. Moreover, WeChat does not involve the evaluation indicator of floating window control operation, thus this evaluation indicator has no score.

**TABLE 7:** WeChat experience evaluation index score.

Evaluation indicators		N	Min	Max	mean	SD
Factor	Perceptibility	22	0.93	3.48	2.37	0.80
	Operability	22	0.32	2.47	1.10	0.60
	Comprehensibility	22	1.34	5.10	3.53	1.19
Code	Q1	22	0.93	3.48	2.37	0.80
	Q2	22	1.00	3.75	2.56	0.86
	Q3	22	0.32	1.79	0.87	0.36
	Q4	22	0.18	1.36	0.52	0.32
	Q10	22	0.06	0.57	0.26	0.18
	Q11	22	0.13	0.66	0.27	0.17
	Q6	22	0.32	2.47	1.10	0.60
	Q7	22	0.06	0.41	0.17	0.10
	Q5	22	0.05	0.31	0.15	0.08
	Q12	22	1.34	5.10	3.53	1.19
	Q9	22	−	−	−	−
	Q8	22	1.40	5.32	3.68	1.24

• Toutiao Big Font Version

It can be discerned from Table 8 that the score for operability within the factor hierarchy of the elderly UX evaluation index of Toutiao Big Font Version is the highest, which is identical to that of the WeChat experience evaluation. The two evaluation indicators, namely completing various gesture operations and having sufficient operation time, in the factor layer of the experience evaluation index of Toutiao Big Font Version have higher scores. Additionally, Toutiao Big Font Version does not encompass the four evaluation indicators of categorizing commonly used application functions, confirming consent for login and usage, confirming mobile phone verification code information, and setting the version for elderly users. For this reason, these four evaluation indicators receive no scores.

**TABLE 8:** Toutiao big font version experience evaluation index score.

Evaluation indicators		N	Min	Max	Mean	SD
Factor	Perceptibility	22	0.99	2.73	1.67	0.45
	Operability	22	0.00	0.58	0.24	0.15
	Comprehensibility	22	1.34	3.31	2.01	0.53
Code	Q1	22	0.99	2.73	1.67	0.45
	Q2	22	1.07	2.94	1.80	0.48
	Q3	22	0.38	2.23	1.20	0.42
	Q4	22	0.30	1.60	0.82	0.36
	Q10	22	–	–	–	–
	Q11	22	–	–	–	–
	Q6	22	–	–	–	–
	Q7	22	–	–	–	–
	Q5	22	0.00	0.58	0.24	0.15
	Q12	22	1.34	3.31	2.01	0.53
	Q9	22	0.06	0.43	0.16	0.12
	Q8	22	1.40	3.46	2.10	0.55

It can thus be observed that the higher ratings of the evaluation items for the UX of these two elderly-oriented mobile applications are predominantly manifested in the completion of various gesture operations and sufficient operation time. Apart from these two evaluation indicators being the objective conditions for obtaining a greater amount of evaluation content data, they also indirectly reveal that the elderly tend to favor simple, rapid, and relatively unrestricted design patterns. Additionally, although the interfaces of both these mobile applications have implemented the design of full-version large fonts, large icons, and large buttons, the elderly users’ evaluation scores for the perceptual aspects of the visual interface, such as fonts, character spacing, colors, icons, and layouts, vary. This might stem from the distinct functional attributes of the two mobile applications, which give rise to differences in usage preferences among the elderly users. For instance, some elderly individuals pay more attention to news hotspots, while others have a higher demand for communication and social interaction. It is comforting to note that the elderly users gave the highest scores for the operability of both mobile applications, indicating their approval of the operation and usage experience of the mobile applications.

### 5.2 Influencing factors of evaluation indexes

Based on the numerical characteristics of the variables, an independent sample *t*-test was conducted. The two demographic variables of the elderly respondents, namely age and gender, were used as independent variables, while the sub-indicators of the three evaluation indicators of the elderly-friendly design of mobile applications, namely perceivability, understandability, and operability, were used as dependent variables. The *F*-values between the two were tested to determine whether there were significant differences, in order to identify whether the evaluation indicators of the elderly-friendly design of mobile applications were affected by age and gender. To eliminate the differences in dimensions, the *Z*-score method was used to standardize the calculated data (Table 9).

**TABLE 9:** Analysis results of influencing factors at the evaluation index layer.

Evaluation indicators	Gender	Age
Perceptibility	0.772	0.127
Operability	0.764	0.043*
Comprehensibility	0.958	0.057

*p* < 0.05, *p < 0.01.

Gender: In the independent sample *t*-test, gender had no significant difference in the three evaluation indicators of the elderly’s experience with mobile applications. The research selects evaluation indicators that focus on the basic experiences of mobile interactive applications (such as interaction Perceptibility, operation smoothness, and functional Comprehensibility), rather than gender-sensitive scenarios, which may mask potential differences. Meanwhile, it is indicated that the evaluation of the basic indicators of mobile interaction applications by elderly men and women is universal.

Age: In the one-way multivariate analysis of variance, age had no significant difference in the evaluation indicators of perceivability (*F* = 2.209, *P* = 0.127) and understandability (*F* = 3.096, *P* = 0.057) of the elderly’s experience with mobile applications, but had a significant difference in the evaluation indicator of operability (*F* = 3.417, *P* = 0.043). Task test time discovery the elderly aged 65–69 achieved the highest scores in the Q5 (The interface setting of the floating window quick control), evaluation index, suggesting that individuals within this age group place greater emphasis on the optimization of mobile application interface design elements. Meanwhile, those aged 75 and above scored the highest in the Q6 (Simple gesture operations) and Q7 (The provision of sufficient operation time) evaluation indices, indicating that this older age group prioritizes the usability and practicality of mobile applications.

### 5.3 Emotion experience evaluation

Currently, the PANAS developed by Watson et al. (1988) is among the widely used tools for measuring emotional wellbeing. In this study, The Cronbach’sα for the PA and NA subscales were 0.77 and 0.70, respectively, demonstrating the reliability and validity of the scales in assessing the emotions of the older adults tested. The PANAS results indicated that the participants did not experience irritability or fear.

The mean score for all positive emotion descriptors was 3.75, while the mean score for all negative emotion descriptors was 1.17. These results suggest that participants’ positive emotions were significantly higher than 3, while their negative emotions were notably lower than 3.

## 6 Conclusion

This study, grounded in cognitive psychology, has comprehensively evaluated the UX factors of elderly mobile applications and established a robust evaluation index system. The research findings underscore the pivotal role of cognitive capabilities and emotional needs of elderly users in shaping their mobile application experience. Specifically, the three main factors identified: perceptibility, operability, and comprehensibility. Directly correspond to the cognitive and perceptual challenges faced by the elderly, as well as their emotional responses to technology use.

From a cognitive psychology perspective, perceptibility is crucial for elderly users due to age-related declines in visual acuity and information processing speed. Larger fonts, clear icon designs, and sufficient color contrast are essential for enhancing the elderly’s ability to perceive information accurately. These design elements not only reduce visual fatigue but also align with the cognitive psychology principle of minimizing cognitive load, allowing users to process information more efficiently.

Operability emerged as another key factor, reflecting the elderly’s motor skill limitations and the need for simple, intuitive interaction patterns. Simple gesture operations and adequate response time are critical in addressing these challenges. Cognitive psychology highlights that the elderly often experience slower reaction times and reduced dexterity, making it essential for mobile applications to provide ample operational time and straightforward interaction methods. This approach aligns with the principles of human-computer interaction, which emphasize the importance of reducing cognitive and physical effort to enhance UX.

Comprehensibility is equally important, as it addresses the cognitive challenges related to memory and information processing. Clear instructions, categorized functions, and confirmation prompts are vital for aiding the elderly’s understanding and recall. Cognitive psychology suggests that the elderly may struggle with encoding and retrieving new information, particularly in unfamiliar digital environments. By incorporating these design elements, mobile applications can support the elderly’s cognitive processes, thereby enhancing their overall UX.

Moreover, the study revealed that demographic factors such as age and gender can influence the evaluation indicators of elderly-friendly design. While gender did not significantly impact the evaluation results, age differences were observed in the operability dimension, indicating that older users may face more pronounced challenges in interaction and navigation. This finding highlights the need for personalized design considerations that account for the diverse cognitive and physical capabilities of elderly users across different age groups.

In conclusion, this research provides a scientific framework for evaluating and improving the UX of elderly mobile applications from a cognitive psychology standpoint. The findings emphasize the necessity of integrating cognitive principles into the design process to enhance the usability, accessibility, and satisfaction of elderly users. Future research should continue to explore the intersection of cognitive psychology and UX design, leveraging emerging technologies and expanding sample sizes to further refine and optimize elderly-friendly mobile applications.

## Data Availability

The original contributions presented in the study are included in the article/Supplementary material, further inquiries can be directed to the corresponding author.

## References

[B1] Ahmad S. Zulkurnain N. N. A. Khairushalimi F. I. (2016). Assessing the validity and reliability of a measurement model in Structural Equation Modeling (SEM). *J. Adv. Math. Comput. Sci.* 15 1–8. 10.9734/BJMCS/2016/25183

[B2] Baharum A. Zain N. H. M. Taharudin A. Hanapi R. Saudi A. Alfred R. (2017). Guidelines of user interface design for elderly mobile applications: A preliminary study. *Asian J. Inform. Technol.* 16 38–44. 10.3923/ajit.2017.38.44

[B3] Barbosa B. Frank V. (2019). *Ageing and Digital Technology: Designing and Evaluating Emerging Technologies for Older Adults.* Cham: Springer.

[B4] Brunzini A. Papetti A. Grassetti F. Moroncini G. Germani M. (2022). The effect of systemic sclerosis on use of mobile touchscreen interfaces: Design guidelines and physio-rehabilitation. *Int. J. Indust. Ergon.* 87:103256. 10.1016/j.ergon.2021.103256

[B5] Comrey A. L. Lee H. B. (1992). *A First Course in Factor Analysis*, 2nd Edn. Hillsdale, NJ: Lawrence Erlbaum.

[B6] Czaja S. J. Boot W. R. Charness N. Rogers W. A. (2019). *Designing for Older Adults: Principles and Creative Human Factors Approaches*, 3rd Edn. Boca Raton, FA: CRC Press/Routledge/Taylor & Francis Group. 10.1201/b22189

[B7] Fischer B. Peine A. Östlund B. (2020). The importance of user involvement: A systematic review of involving older users in technology design. *Gerontologist* 60 e513–e523. 10.1093/geront/gnz163 31773145 PMC7491439

[B8] Forlizzi J. Ford S. (2000). The building blocks of experience: An early framework for interaction designers. *Proc. Des. Interact. Syst.* 6 419–423. 10.1145/347642.347800

[B9] Fornell C. Larcker D. F. (1981). Structural equation models with unobservable variables and measurement error: Algebra and statistics. *J. Mark. Res.* 18 382–388. 10.1177/002224378101800313

[B10] Ghorbel F. Métais E. Ellouze N. Hamdi F. Gargouri F. (2017). “Towards accessibility guidelines of interaction and user interface design for Alzheimer’s Disease patients,” in *Proceedings of the International Conference on Advances in Computer-Human Interaction*, (Porto: IARIA).

[B11] Hair J. F. (2009). *Multivariate Data Analysis*, 7th Edn. New York, NY: Pearson.

[B12] Harada S. Sato D. Takagi H. Asakawa C. (2013). Characteristics of elderly user behavior on mobile multi-touch devices. *Hum.-Comput. Int.* 2013, 323–341. 10.1007/978-3-642-40498-6_25

[B13] Hassenzahl M. (2004). The interplay of beauty, goodness, and usability in interactive products. *Hum. Comput. Interact.* 19 319–349. 10.1207/s15327051hci1904_2

[B14] Hassenzahl M. (2010). *Experience Design: Technology for All the Right Reasons.* San Rafael, CA: Morgan & Claypool.

[B15] Ho H. H. Tzeng S. Y. (2021). Using the Kano model to analyze the user interface needs of middle-aged and older adults in mobile reading. *Comput. Hum. Behav. Rep.* 3:100074. 10.1016/J.CHBR.2021.100074

[B16] Holzinger A. Searle G. Nischelwitzer A. (2007). “On some aspects of improving mobile applications for the elderly” in *Coping with Diversity in Universal Access, Research and Development Methods in Universal Access*, ed. Stephanidis C. (Cham: Springer), 4544 923–932. 10.1007/978-3-540-73279-2_103

[B17] Hou G. Hu Y. (2023). Designing combinations of pictogram and text size for icons: Effects of text size, pictogram size, and familiarity on older adults’visual search performance. *Hum. Fact.* 65 1577–1595. 10.1177/00187208211061938 34970924

[B18] Hsieh M. H. Ho C. H. Lee I. C. (2022). Effects of smartphone numeric keypad designs on performance and satisfaction of elderly users. *Int. J. Ind. Ergon.* 87:103236. 10.1016/j.ergon.2021.103236

[B19] Hu F. Feng Z. Y. Liu D. C. Wang W. (2020). User experience design: From concept to method. *Pack. Eng.* 16 51–63. 10.19554/j.cnki.1001-3563.2020.16.009

[B20] Huang T. Wang G. Huang C. W. (2024). What promotes the mobile payment behavior of the elderly?. *Human. Soc. Sci. Commun.* 11 1–14. 10.1057/s41599-024-04031-z

[B21] Hunsaker A. Hargittai E. (2018). A review of Internet use among older adults. *N. Media Soc.* 20 3937–3954. 10.1177/1461444818787348

[B22] Iancu I. Bogdan I. (2020). Designing mobile technology for elderly. A theoretical overview. *Technol. Forecast. Soc. Change* 155:119977. 10.1016/j.techfore.2020.119977

[B23] Iqbal S. Irfan M. Ahsan K. Hussain M. A. Shiraz M. Hamdi M. (2020). A novel mobile wallet model for elderly using fingerprint as authentication factor. *IEEE Access* 8 177405–177423. 10.1109/ACCESS.2020.3025429

[B24] Jimoyiannis A. Gravani M. N. (2014). Digital literacy in a lifelong learning programme for adults: Educators’ experiences and perceptions on teaching practices. *Int. J. Digital Literacy Digital Compet.* 1 40–60. 10.4018/jdldc.2010101903

[B25] Kim M. J. Lee C. K. Contractor N. S. (2019). Seniors’ usage of mobile social network sites: Applying theories of innovation diffusion and uses and gratifications. *Comput. Hum. Behav.* 90 60–73. 10.1016/J.CHB.2018.08.046

[B26] Law E. L. C. Schaik V. P. Roto V. (2014). Attitudes towards user experience (UX) measurement. *Int. J. Hum. Comput. Stud.* 72 526–541. 10.1016/j.ijhcs.2013.09.006

[B27] Leeson G. W. (2018). Global demographic change and the case of low fertility. *Populat. Horiz.* 15 1–6. 10.2478/pophzn-2018-0007

[B28] Li C. Lee C. F. Xu S. (2020). Stigma threat in design for older adults: Exploring design factors that induce stigma perception. *Int. J. Des.* 14 51–64

[B29] Li Q. (2019). *User Modelling for Older Adults’ Mobile Interaction Behaviour: Evaluation of User Characteristics, Task Demands And Interface Design.* Hong Kong: Hong Kong Polytechnic University.

[B30] Li Q. Luximon Y. (2018). Understanding older adults’ post-adoption usage behavior and perceptions of mobile technology. *Int. J. Des.* 12 93–110.

[B31] Lin C. J. Ho S. H. (2020). The development of a mobile user interface ability evaluation system for the elderly. *Appl. Ergon.* 89 1–19. 10.1016/j.apergo.2020.103215 32791347

[B32] Mahlke S. (2005). *Understanding Users’ Experience of Interaction.* Berlin: Berlin University of Technology.

[B33] McLaughlin A. Pak R. (2020). *Designing Displays for Older Adults*, 2nd Edn. Boca Raton, FL: CRC Press, 10.1201/9780429439674

[B34] Menghi R. Ceccacci S. Gullà F. Cavalieri L. Germani M. Bevilacqua R. (2017). “How older people who have never used touchscreen technology interact with a tablet,” in *Proceedings of the IFIP TC13 International Conference on Human-Computer Interaction*, eds Bernhaupt R. Dalvi G. Joshi A. Balkrishan K. O’Neill J. Winckler M. (Cham: Springer), 117–131.

[B35] Mitchell U. A. Chebli P. G. Ruggiero L. Muramatsu N. (2019). The digital divide in health-related technology use: The significance of race/ethnicity. *Gerontologist* 59 6–14. 10.1093/geront/gny138 30452660

[B36] Mitzner T. L. Boron J. B. Fausset C. B. Adams A. E. Charness N. Czaja S. J. (2010). Older adults talk technology: Technology usage and attitudes. *Comput. Hum. Behav.* 26 1710–1721. 10.1016/j.chb.2010.06.020 20967133 PMC2956433

[B37] Nielsen J. (1994). *Usability Engineering.* Burlington, MA: Morgan Kaufmann.

[B38] Nilsson L. G. (2003). Memory function in normal aging. *Acta Neurol Scand. Suppl.* 179 7–13. 10.1034/j.1600-0404.107.s179.5.x 12603244

[B39] Park J. Han S. H. Kim H. K. Oh S. Moon H. (2013). Modeling user experience: A case study on a mobile device. *Int. J. Indust. Ergon.* 43 187–196. 10.1016/J.ERGON.2013.01.005

[B40] Parra-Rizo M. A. Vásquez-Gómez J. Álvarez C. Diaz-Martínez X. Troncoso C. Leiva-Ordoñez A. M. (2022). Predictors of the level of physical activity in physically active older people. *Behav. Sci.* 12:331. 10.3390/bs12090331 36135135 PMC9495331

[B41] Perrig S. A. Aeschbach L. F. Scharowski N. Felten N. V. Opwis K. Brühlmann F. (2024). Measurement practices in user experience (UX) research: A systematic quantitative literature review. *Front. Comput. Sci.* 6:1368860. 10.3389/fcomp.2024.1368860

[B42] Petrovčič A. Taipale S. Rogelj A. Dolničar V. (2018). Design of mobile phones for older adults : An empirical analysis of design guidelines and checklists for feature phones and smartphones. *Int. J. Hum. Comput. Interact.* 34 251–264. 10.1080/10447318.2017.1345142

[B43] Pucillo F. Cascini G. (2014). A framework for user experience, needs and affordances. *Design Stud.* 35 160–179. 10.1016/j.destud.2013.10.001

[B44] Salazar-Cardona J. A. Cano S. Gutiérrez-Vela F. L. Arango J. (2023). Designing a Tangible User Interface (TUI) for the elderly based on their motivations and game elements. *Sensors* 23:9513. 10.3390/s23239513 38067886 PMC10708562

[B45] Shevlin M. Miles J. N. (1998). Effects of sample size, models pecification and factor loadings on the GFI in confirmatory factor analysis. *Pers. Individ. Differ.* 25 8–90. 10.1016/S0191-8869(98)00055-5

[B46] Stephanie Morey W. R. Laura H. Barg-Walkow L. (2017). Managing heart failure on the go: Usability issues with mhealth apps for older adults. *Proc. Hum. Fact. Ergon. Soc. Annu. Meet.* 61 1–5. 10.1177/1541931213601496

[B47] Watson D. Clark L. A. Tellegen A. (1988). Development and validation of brief measures of positive and negative affect: The PANAS scales. *J. Pers. Soc. Psychol.* 54 1063–1070. 10.1037//0022-3514.54.6.1063 3397865

[B48] Wildenbos G. A. Jaspers M. W. M. Schijven M. P. Dusseljee-Peute L. W. (2019). Mobile health for older adult patients: Using an aging barriers framework to classify usability problems. *Int. J. Med. Inform.* 124 68–77. 10.1016/j.ijmedinf.2019.01.006 30784429

