# Supplementary material for: The psychological influencing factors for the elderly in using mobile applications and the mechanism of user experience
Source: Front Psychol. 2025 Jul 18;16:1609302. doi: 10.3389/fpsyg.2025.1609302 (PMC12313668; doi:10.3389/fpsyg.2025.1609302)
Supplement: Supplementary file 1 [file Table_1.docx]

## 移動應用程序適老化改造之滿意度調查

Survey on the satisfaction of mobile application for aging modification

| 敬愛的長者，您好！  感謝您參與本調查  隨著數字生活的不斷提升，老年群體對APP使用需求愈加增長。當前，許多APP面向社會各類使用人群，對於老年群體特徵與需求缺乏一定考慮。因此，探討老年人對現有APP的使用滿意度調查是相當重要的議題。我們希望借助您過往經驗和真實感受，提供您的寶貴意見，本問卷題項沒有正確與錯誤之分別，調查結果僅用於學術研究，請安心填寫，忠心期盼您協助與填寫本問卷。  请您仔细阅读问卷中的题目，选择相应的方框打勾即可。再次感謝您的協助與支持。  福建理工大學  Dear elder, hello!  Thank you for participating in this survey With the continuous improvement of digital life, the elderly group's demand for APP use is increasing. At present, many apps are oriented to all types of users in society, and lack certain consideration for the characteristics and needs of the elderly group. Therefore, it is a very important topic to explore the elderly's satisfaction survey on the use of existing apps. We hope to provide you with valuable opinions based on your past experience and real feelings. There is no difference between right and wrong in this questionnaire, and the survey results are only for academic research. Please feel free to fill in this questionnaire. Please read the questions in the questionnaire carefully and select the corresponding boxes to tick. Thank you again for your assistance and support.  Fujian University of Science and Technology |
| --- |

**第一部分：基本情況**

**Part Ⅰ: Basic situation**

1.您的年齡？

□60-64歲 □65-70歲以上 □70歲以上

1.What is your age?

□60-64 years old □65-70 years old or older □70 years old or older

2.您的性別? (訪問員可讀出以下的選擇)

□男性 □女性

1. What is your gender? (The visitor can read the following selection)

□ Male □ female

3.您的居住的城市？

□直轄市 □省會城市 □中小城鎮 □其他

1. What city do you live in?

□ Municipalities directly under the Central Government □ Provincial capitals □ Small and medium-sized towns □ Others

4.您已使用智能手機多久時間？

□不到一年 □一年至三年 □三年以上

1. How long have you been using your smartphone?

□ Less than one year □ One year to three years □ More than three years

5. 您每天使用智能手機累計多久時間？

□一個小時內 □一至三個小時 □三個小時至六個小時以上

□六個小時以上

1. How much time do you spend using your smartphone every day?

□ Within an hour □ 1 to 3 hours □ 3 hours to more than 6 hours □ More than six hours

6.您是否在使用APP時尋求過他人幫助?

□是 □否

1. Have you asked others for help when using the APP?

□ Yes □ no

7. 您有在下列場景中因不會使用移動應用程序（APP）而影響到日常生活嗎？

□出示健康碼 □線上繳納水電等生活費用

□銀行業務辦理 □醫院掛號手機 □手機支付 □其他 □無

1. In any of the following scenarios, have you affected your daily life by not using mobile applications (apps)?

□ Show health code □ Pay living expenses such as water and electricity online

□ Banking transaction □ Hospital registered mobile phone □ Mobile phone payment □ Other □ None

8.按照使用頻率，選擇三款您每天使用最頻繁的移動應用程序（APP）

（請選擇2-3項）：

□社交類（微信等） □新聞類APP（今日頭條等）

□短視頻APP(抖音等) □視頻類APP（騰訊視頻等）

□金融支付類（支付寶等） □購物類（淘寶等）

□外賣類APP（美團等） □看病、掛號類APP（丁香醫生等）

□導航類APP（高德地圖等） □以上都不是

1. According to the frequency of use, select the three mobile apps that you use most frequently every day. (Please select 2-3 items) :

□ Social (wechat, etc.) □ News APP (Toutiao, etc.) □ Short video APP(Tiktok, etc.) □ Video APP(Tencent Video, etc.) □ Financial payment (Alipay, etc.) □ Shopping (Taobao, etc.) □ Takeaway APP (Meituan, etc.) □ Doctor, registration APP (Dingxiang Doctor, etc.) Navigation APP (Gaode Map, etc.) □ None of the above

9.您下載新APP的原因是什麼？

□他人推薦 □廣告推廣 □生活需要 □強制要求

1. What is your reason for downloading a new APP?

□Recommended by others □Advertising □Life needs

□Mandatory requirements

10.您是否使用各類APP適老版本?

□從未使用過 □使用過，但未持續使用 □使用過，並持續使用

1. Do you use older versions of various apps?

□ Never used □ Used, but not continuously used □ Used, and continue to use

**第二部分：請參照您目前使用的APP情況，以您真實感受與使用經歷對APP顯示界面、使用操作、提示機制、技術相容、安全風險這五個方面進行滿意度評價。**

**Part II: Please refer to the situation of the APP you are currently using, and evaluate the satisfaction of the five aspects of APP display interface, operation, prompt mechanism, technical compatibility and security risk based on your real feelings and experience.**

1. 您對APP使用過程時的顯示界面設計滿意度如何？

11. How satisfied are you with the display interface design during APP use?

| NO. | - problem | 滿意  satisfaction | 比較滿意Relatively satisfied | 一般  Normal | 比較不滿意  Less satisfied | 不滿意  Not satisfied |
| --- | --- | --- | --- | --- | --- | --- |
| T11-01 | 界面字體大小適中  The font size is moderate |  |  |  |  |  |
| T11-02 | 界面文字與文字的距離適中  The distance between text and interface text is moderate |  |  |  |  |  |
| T11-03 | 圖示與文字呈現清晰明瞭  Illustrations and text are presented clearly and clearly |  |  |  |  |  |
| T11-04 | 出現錯誤信息與重要信息會有色彩區別提示  Error messages and important messages will be color-coded |  |  |  |  |  |
| T11-05 | 使用時與其他APP綁定，實現授權登錄  Binding with other apps to achieve authorized login |  |  |  |  |  |
| T11-06 | 使用時採用本機號碼一鍵登錄  Use the local number to log in with one key |  |  |  |  |  |
| T11-07 | 使用時採用手機號碼加短信驗證碼登錄  Use the mobile phone number and SMS verification code to log in |  |  |  |  |  |

1. 您對APP使用操作的滿意度如何？

12. How satisfied are you with the APP operation?

| NO. | - problem | 滿意  satisfaction | 比較滿意Relatively satisfied | 一般  Normal | 比較不滿意  Less satisfied | 不滿意  Not satisfied |
| --- | --- | --- | --- | --- | --- | --- |
| T12-01 | 能完成觸碰、拉動、點擊的手勢操作Can complete touch, pull, click gesture operation |  |  |  |  |  |
| T12-02 | 使用APP可以有充足的操作時間Use the APP to have sufficient operation time |  |  |  |  |  |
| T12-03 | APP存在浮窗快捷按鍵 Floating window shortcut button exists in APP |  |  |  |  |  |

1. 您對APP使用過程時的提示機制滿意度如何？

13. How satisfied are you with the prompt mechanism during APP use?

| NO. | - problem | 滿意  satisfaction | 比較滿意Relatively satisfied | 一般  Normal | 比較不滿意  Less satisfied | 不滿意  Not satisfied |
| --- | --- | --- | --- | --- | --- | --- |
| T13-01 | 支持適老版本切換提示  Support for older version switching prompt |  |  |  |  |  |
| T13-02 | 常用功能具有醒目的引導入口Common features feature an eye-catching bootstrap entry |  |  |  |  |  |
| T13-03 | 通過搜索可直達所需功能Search to get to what you need |  |  |  |  |  |

1. 您對APP相容技術的滿意度如何？

14. How satisfied are you with APP-compatible technologies?

| NO. | - problem | 滿意  satisfaction | 比較滿意Relatively satisfied | 一般  Normal | 比較不滿意  Less satisfied | 不滿意  Not satisfied |
| --- | --- | --- | --- | --- | --- | --- |
| T14-01 | 手機APP可在其他終端產品上使用，如平板、手錶、電腦等設備  Mobile APP can be used on other terminal products, such as tablets, watches, computers and other devices |  |  |  |  |  |
| T14-02 | 未更新到最新版本，仍可繼續使用現有版本If you have not updated to the latest version, you can continue to use the existing version |  |  |  |  |  |

1. 您對APP防範安全風險的滿意度如何？

15. How satisfied are you with the APP's ability to prevent security risks?

| NO. | - problem | 滿意  satisfaction | 比較滿意Relatively satisfied | 一般  Normal | 比較不滿意  Less satisfied | 不滿意  Not satisfied |
| --- | --- | --- | --- | --- | --- | --- |
| T15-01 | 防止下載或支付的風險  Prevents the risk of downloading or paying |  |  |  |  |  |
| T15-02 | 防止個人信息隱私洩露Prevent privacy disclosure of personal information |  |  |  |  |  |
| T15-03 | 發送安全風險提醒信息Send security risk alerts |  |  |  |  |  |
| T15-04 | 無廣告彈窗與廣告視頻No AD pop-ups and AD videos |  |  |  |  |  |
